# Surveillance of moderate-size aneurysms of the thoracic aorta

**DOI:** 10.1186/s13019-015-0220-2

**Published:** 2015-02-06

**Authors:** Allison J McLarty, Muath Bishawi, Suresh Baba Yelika, A Laurie Shroyer, Jamie Romeiser

**Affiliations:** 1 Northport VA Medical Center, Northport, NY USA; 2 Departments of Surgery and Preventive Medicine, Stony Brook University School of Medicine, Stony Brook, NY USA; 3 Division of Cardiovascular and Thoracic Surgery, Department of Surgery, Duke University, Durham, NC USA; 4 Cardiothoracic Surgeon, Department of Surgery, Northport VA Medical Center, Northport, NY USA; 5 Department of Surgery, Division of Cardiothoracic Surgery, Stony Brook University, Stony Brook, NY USA

**Keywords:** Moderate thoracic aortic aneurysms, Surveillance strategy, Radiation exposure

## Abstract

**Background:**

There are no evidence based guidelines for the surveillance of patients with moderate-sized (<5 cm) thoracic aortic aneurysms (MTAA), who do not warrant surgical intervention. The purpose of this study was to review the MTAA patient surveillance strategy used currently at the Northport Veterans Affairs Medical Center, to assess outcomes over time and accrue data to develop guidelines to optimize MTAA patients’ follow-up.

**Methods:**

The study group included veterans referred to the Thoracic Surgery clinic for the management of moderate-sized (<5 cm) thoracic aortic aneurysms (MTAA) not warranting immediate surgical repair.

As a pilot study, all MTAA patients’ charts from 2005–2013 were reviewed to describe imaging practices and evaluate patient-specific long-term outcomes. An adverse composite endpoint was defined if a patient’s aneurysm grew substantially (≥0.5 cm/year or reached 5.5 cm) or a MTAA-related event (surgery or death) occurred.

Additionally, number of CT scans obtained during the follow up period were documented.

**Results:**

For 110 MTAA patients, the average presenting index size was 4.45 ± 0.4 cm with average growth of 0.04 cm total (0.03 cm/year). Fourteen (13%) patients met the adverse composite endpoint, with no MTAA-related deaths. Patients achieving the adverse composite endpoint had higher index sizes (4.81 vs. 4.40 cm, p = 0.001) and higher average growth rates as compared to non-endpoint patients (0.16 vs. 0.01 cm, p = 0.0009). Optimizing the negative likelihood ratio defined a new “not-at-risk” population with aneurysm index size < 4.3 cm. A shorter time to adverse event for “at-risk” patients was found versus “not-at-risk” patients (p = 0.02). On average, there were 4.8 CT scans/patient and estimated cumulative radiation dose of 34 mSv/patient. Only one “not-at-risk” patient had substantive MTAA growth (≥0.5 cm/year) over the 8 year follow-up period.

**Conclusion and relevance:**

Annual imaging of MTAA “not-at-risk” patients appears unwarranted, resulting in potentially excessive radiation exposure. Although additional research is necessary for validation, longer surveillance imaging intervals (beyond one year) seem appropriate for MTAA patients presenting with < 4.3 cm index aneurysms.

## Background

Thoracic aortic aneurysms account for almost 50,000 deaths annually in the United States [1]. Death is due to rupture or dissection and is strongly linked to the size of the aneurysm [2]. Recent Guidelines for the Diagnosis and Management of Patients with Thoracic Aortic Disease represent the efforts of multiple professional societies (ACCF/AHA/AATS/ACR/ASA/SCA/SCAI/SIR/STS/SVM) to develop evidence based recommendations for the management of all aspects of thoracic aortic disease in order to improve patient outcomes [3]. The guidelines were prepared after rigorous and careful review of the evidence by multiple experts in the field representing cardiologists, radiologists, vascular surgeons and cardiothoracic/aortic surgeons.

Current guidelines recommend surgical intervention for ascending thoracic aortic aneurysms when they are 5 cm in size for patients with genetic predisposition to rupture/dissection such as the connective tissue disorder Marfan’s syndrome, or 5.5 cm in size for non-Marfan patients [3]. Similarly the recommendations are to surgically intervene when a descending thoracic aortic aneurysm is 5.5 cm for a Marfan or 6 cm for a non-Marfan patient [2-6]. These guidelines are based on data showing increased mortality for these patients. Thus, the risk of rupture-related complication exceeds the risk of surgery-related complications for this high risk patient sub-group - rendering an operation as the safer strategy. For *moderate-size (defined as ≤ 4.9 cm) thoracic aortic aneurysms (MTAA)*, however, the committee concluded that the evidence is severely lacking to establish recommendations. These MTAA patients are generally not referred for surgery- but the appropriate frequency and mode of surveillance are unclear [3].

The computed tomography (CT) scan is currently the most commonly used form of aortic imaging, but carries with it the risk of ionizing radiation. Only weak (level C) evidence suggests the use of magnetic resonance imaging for these patients instead of computed tomographic imaging in an effort to reduce such harmful exposure [3]. Echocardiography is used sporadically, as clinicians have concerns about the reliability and accuracy of echo-based TAA aneurysm sizes [7].

In our Thoracic Surgical Clinic we see many patients with MTAA. Current management is guided by best clinical judgment. There is no data to guide surveillance of these patients. Given the scarcity of reported data on MTAA patients, the aim of our pilot study was to evaluate the natural history of a cohort of our MTAA patients, to review the surveillance strategy used, and to report on our patients’ long-term outcomes.

## Methods

At the Northport VA Medical Center, Institutional Review Board (IRB) and Research and Development (R&D) Committee approvals were obtained prior to initiating the retrospective medical chart reviews for our pilot study (IRB# 00408). For all MTAA patients seen during the period from 2005 to 2013 in our VA Thoracic Surgery Clinic, the patients’ medical records were carefully reviewed and data extracted related to TAA rupture-related risk factors (for example hypertension, cigarette smoking, see Table 1), surveillance imaging approaches used (including detailed radiologic findings by image type), as well as patient-specific short-term and longer-term clinical outcomes.

**Table 1:** **Demographics of the patient cohort**

Risk Factor	N (%)
Age (Mean, Std)	70 ± 9
Body Mass Index [BMI] (Mean, Std)	31.6 ± 6
Systolic Blood Pressure (Mean, Std)	132 ± 14
Diastolic Blood Pressure (Mean, Std)	76.3 ± 12
Hypertension	89 (81%)
Diabetes	21 (19%)
Chronic Kidney Disease [CKD]	8 (7%)
Cerebrovascular Disease [CVD]	9 (8%)
Peripheral Vascular Disease [PVD]	5 (5%)
Prior Myocardial Infarction [MI]	19 (17%)
Previous Percutaneous Coronary Intervention [PCI]	19 (17%)
History of Coronary Artery Bypass Graft [CABG] Procedure	17 (15%)
Smoking Status	
Last smoked < 2 weeks of surgery	14 (13%)
Last smoked > 1 year before surgery	68 (62%)
>3 months to < 1 year before surgery	2 (1.8%)
Never Smoked	17 (15.6%)
Unknown	8 (7%)
Hyperlipidemia	65 (59%)
Use of Diuretic Medications	35 (32%)
Use of ACE or ARB Medications	52 (47%)
Use of Beta-blocker [BB] Medication	45 (41%)
Use of Lipid Lowering Agent [LLA] Medication	57 (52%)

To ensure the accuracy of all chart abstractions performed, a small set of patient records were pre-tested by two independent data collectors (SB – research resident, and AM – senior surgical faculty attending) After comparing these pre-test charts, the data collection forms were modified to assure accuracy for all future data captured. All remaining data was collected by one person (SB) for internal consistency.

### Study participants

Starting in December 2005, the records for all of Northport VA Thoracic Surgery Clinic patients were screened for potential inclusion in the study. Exclusion criteria included: (a) coarctation of the aorta; (b) any genetic disorder (i.e. Marfan syndrome, Ehlers-Danlos syndrome, vascular form, Turner syndrome, Loeys-Dietz syndrome, Familial thoracic aortic aneurysm and dissection syndrome, Inflammatory vasculitides, Takayasu arteritis, Giant cell arteritis, and Behçet arteritis); (c) bicuspid aortic valvular disease; or (d) a missing index CT scan (i.e., such that the size of the aneurysm could not be accurately ascertained at baseline).

### Primary end point

Patients were considered to have met the study’s primary adverse composite end point if one of the following outcomes occurred at any point in time during the follow up: 1) the size of the aneurysm grew at least 0.5 cm/year; 2) the aneurysm grew to be at least 5.5 cm; 3) the patient was offered surgery for the aneurysm; or 4) there was an aneurysm-related death. Due to accuracy and ready accessibility, CT scans have traditionally been the diagnostic test of choice to measure aneurysm size. For our patient population, therefore, changes over time in serial CT images were used to evaluate the achievement of the primary study endpoints.

### Analysis plan

A descriptive analysis of patient demographics, risk factors and CT scan data was conducted and reported as mean +/− SD or n (%) when indicated. In order to normalize growth measurements within each patient, total growth per year for each patient was defined as the sum of all growth measurements over the total time followed (calculated in days, divided by 365). First year growth rate was defined as the sum of all growth measurements within the first 390 days over the sum of all time measurements within the first 390 days, divided by 365. Normalized for a year period appropriately, it should be noted that 390 days was used to capture a greater number of patients’ first year CT aneurysm size measurements.

A Kaplan-Meier curve documented the time from initial MTAA diagnosis to time of any adverse composite event; plotted for all patients, as well as for patient risk sub-groups. Univariate screening was performed to identify risk factors associated with time to the adverse composite endpoint using Kaplan-Meier (for categorical predictors) or Cox Regression (for continuous predictors). Interactions between patient characteristics and aneurysm-related risk variables were also tested. Two methods were used to identify a categorical cut-off value for an index size measurement that might indicate a higher “at-risk” patient sub-population. First, a visual inspection of the frequency distribution of initial index size was used to identify any natural cut-point appearing in the data [8,9]. Then, a negative likelihood ratio (NLR) analysis was used to validate this cut-point and identify the lowest aneurysm index size (as the new “threshold”) that would differentiate patient risk sub-groups related to their potential for achieving the study’s primary, adverse, composite end point [10]. Otherwise stated, a negative likelihood ratio is the probability of a negative test result given the presence of the disease (i.e. false negative probability), divided by the probability of a negative test result given the absence of disease (i.e. true negative probability). In this case, disease equates to meeting the study’s adverse end point criteria, and a low NLR would indicate a low chance of an individual who was classified as “low-risk” to meet the adverse endpoint. Negative likelihood ratios are known for ruling conditions out, and thus an appropriate measure to use when defining a threshold for possible reduction of surveillance. All calculations were performed at the p = 0.05 level using SAS©9.2 software, SAS Institute Inc., Cary NC. To report on radiation exposure, based on the most recent published literature, a CT scan of the chest was estimated to provide on average 7 mSV of radiation exposure per imaging session [11].

## Results

A total of 156 Thoracic Surgery clinic patient records were identified for chart review. After preliminary record screening, 44 patients were excluded for not having either a baseline CT scan (to establish initial aneurysm size) or having at least one additional CT scan during the study follow-up period. Additionally, the patients presenting with greater than mild aortic insufficiency (AI) were also excluded (n = 2). Thus, the total number of patient records included in this pilot study’s medical chart review was 110. The anatomical distribution of these included 94 (86%) in the ascending aorta, 8 (7%) in the ascending and arch, and 8(7%) in the descending aorta.

The average patient age was 70 ± 9 years; 80% of the patients were diagnosed as hypertensive (Table 1). A history of tobacco use was very common in this population, with “never smokers” representing only 15.6% of the population.

The average time from the index CT scan to the last follow-up CT scan was 3.23 years (STD: 2.23, RANGE: 0.11 - 9.88). The mean presenting aneurysm index size was 4.45 cm (STD: 0.37, RANGE: 3.8 - 5.4). During the 8 year follow-up period, there was very little aneurysm growth observed (on average, 0.04 cm total per patient, 0.03 cm per patient per year) (Tables 2 and 3).

**Table 2:** **Univariate findings for patients with and without an adverse composite endpoint**

	All patients	Without any adverse composite endpoints	With any adverse composite endpoints	p-value
Number of Patients	110(100%)	96 (87.27%)	14 (12.73%)	--
Total Years Followed (Time from First to Last CT Scan)	3.23 (2.23)	3.04 (2.19)	4.58 (2.12)	0.01
Patient Characteristics				
Age (Mean, STD)	70.02 (9.46)	69.59 (9.42)	72.93 (9.59)	0.22
Proportion of Patients with Hypertension	89 (80.91%)	78 (81.25%)	11 (78.57%)	0.73
Aortic Aneurysm [AA] Measurement Description				
Index Size (Mean, STD)	4.45 (0.37)	4.40 (0.33)	4.81 (0.40)	0.001
Final Size (Mean, STD)	4.49 (0.46)	4.40 (0.35)	5.17 (0.51)	<0.0001
Total Average Growth (Mean, STD)	0.04 (0.29)	−0.004 (0.23)	0.36 (0.34)	0.0009
Total Average Growth Per Year (Mean, STD)	0.03 (0.24)	0.01 (0.22)	0.16 (0.42)	0.017
Average First Year Growth (Mean, STD)	0.02 (0.24)	0.02 (0.20)	0.01 (0.47)	0.15
First Year Growth Rate (Mean, STD)	−0.33 (3.51)	0.008 (0.34)	−2.63 (9.79)	
First Year Growth Rate Alternative Calculation (Mean, STD)	−0.009 (0.42)	−0.01 (0.32)	−0.001 (0.85)	0.133

**Table 3:** **Detailed description of 14 individuals with an adverse composite endpoint**

Adverse event descriptions	Patient count	Criteria #1: growth of 0.5/yr	Criteria #2: any size ≥ 5.5	Criteria #3: offered surgery	All categories
**Criteria #1: Growth of 0.5/yr**	**12**	7	3	0	2
**Criteria #2: Any size ≥ 5.5**	**7**	3	2	0	2
**Criteria #3: Offered surgery**	**2**	0	0	0	2

Fourteen patients (13%) met this pilot study’s adverse composite endpoint. Of these patients: 7 had a growth of at least 0.5 cm/year, 2 had a follow up scan of at least 5.5 cm, 3 had both a growth of at least 0.5 cm/year and a follow up scan of at least 5.5 cm, and 2 were offered surgery in addition to having growth of at least 0.5 cm/year and a follow up scan of at least 5.5 cm. (Table 3). During the follow-up period, there were no aneurysm-related deaths.

In general, MTAA patients who achieved the adverse composite endpoint had higher index size (4.81 vs. 4.40 cm, p = 0.001) and a higher growth rate per year as compared to the “non-endpoint” patients (0.16 vs. 0.01 cm, p = 0.0009). Risk factors such as smoking, age, or hypertension appeared very similar between these two patient sub-groups (p > 0.2).

**Figure 1:**
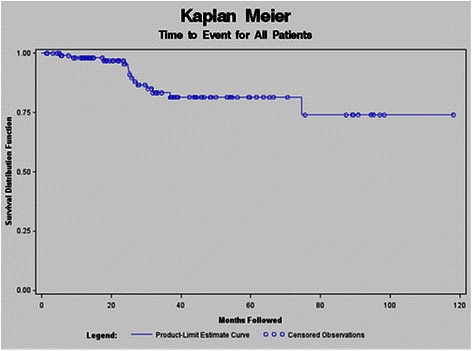
**Kaplan Meier for all patients followed.** A Kaplan Meier curve for all patients, evaluating the time from initial diagnosis to the time of adverse composite endpoint was developed.

A Kaplan Meier curve for all patients, evaluating the time from initial diagnosis to the time of adverse composite endpoint was developed (Figure 1). Univariate screening for risk factors predicting time to event were calculated. The only variable to achieve significance in the univariate time to event screening was aneurysm index size (p = 0.002).

**Figure 2:**
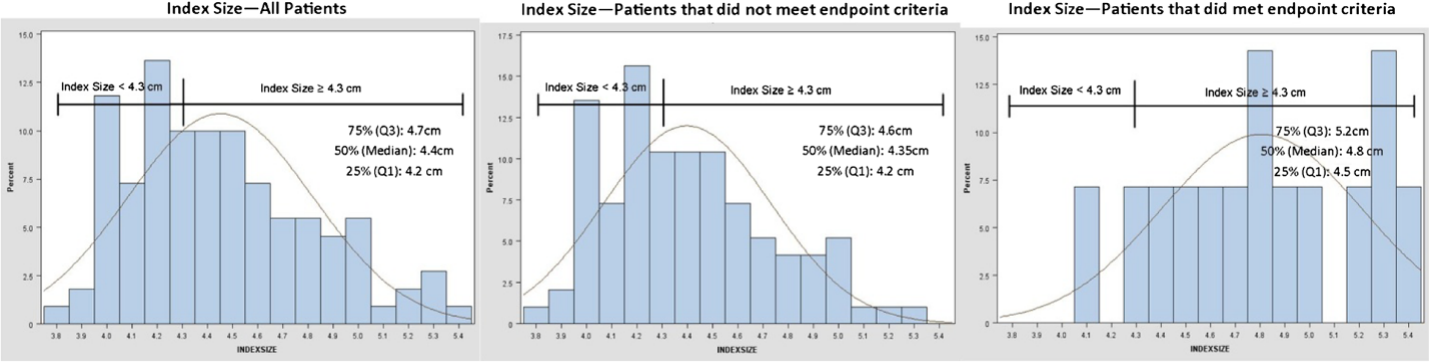
**Frequency distributions for index size measurements.** Data visualization for initial index size showing a natural cut-point for all patients, as well as for subgroups that met or did not meet the study endpoint.

To further explore a potential index size threshold that might be associated with an adverse composite endpoint occurrence, the aneurysm index size was graphically displayed for the sub-groups with and without an adverse composite endpoint (Figure 2). Based on visual inspection, a natural cut point in the data appeared to be located around 4.3 cm. A negative likelihood ratio analysis (NLR) was used to validate this cut point; the smallest NLR was achieved using ≥ 4.3 cm as the threshold measure to differentiate “at-risk” versus “not-at-risk” patients for an adverse, composite event. It should be noted that the NLR = 0.18 generally indicates only mild to moderate refinement in the diagnostic process; thus, this threshold may likely be useful for clinical decision-making purposes when combined with other relevant diagnostic test information (i.e., patient history) and/or expert clinician judgment.

**Figure 3:**
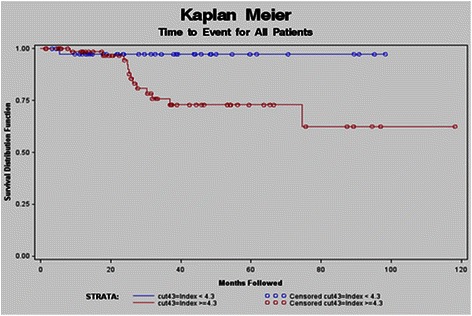
**Kaplan Meier index size value measurement ≥ 4.3 cm.** Using this novel “not-at-risk” cut-off value, time to event was graphed showing a significant difference in time to event for those with a ≥ 4.3 cm as compared to those with a < 4.3 cm index aneurysm (p = 0.02).

Using this novel “not-at-risk” cut-off value, time to event was graphed (Figure 3), showing a significant difference in time to event for those with a ≥ 4.3 cm as compared to those with a < 4.3 cm index aneurysm (p = 0.02). Using the index size threshold of > 4.3 cm, moreover, there was only one “not-at-risk” patient with an index aneurysm size under this 4.3 cm threshold that achieved any study adverse endpoint. For this specific patient, the first endpoint (aneurysm growth ≥ 0.5 cm/year) was met within 120 days following initial diagnosis; but no other study adverse endpoints (e.g., total growth threshold, TAA-related surgery, or TAA-related death) were documented.

To evaluate the cumulative radiation exposure, the timing and findings for all images were compiled. For the 110 patients, there were a total of 525 CT scans performed: 434 CT scans for patients that did not achieve a study endpoint and 91 CT scans for patients achieving any study endpoint. On average, there were 4.8 scans for all patients; 4.5 scans for patients with no endpoint achieved versus 6.5 scans for patients reaching any component of the adverse composite endpoint. Although aneurysm size was recorded on all scans, approximately half (n = 262/525; generally the older scans) of the historical CT scans did not have radiation dosage information recorded. As detailed radiation exposure data were not uniformly available for all scans, an estimated 7 mSv radiation dose per scan was used to calculate the total cumulative radiation dose per person [11]. With an estimated cumulative effective radiation dose of 34 mSv/patient, the radiation exposure to our patients was identified to be an important risk related to our historical surveillance monitoring practices.

## Discussion

Aortic aneurysms are of concern because of their potential risk of death. The likelihood of a mortal event occurring has been linked historically to aneurysm size. Elegant natural history studies from the 70s and 80s demonstrated a significant increased incidence of mortality at size greater than 6 cm for the ascending aorta and 7 cm for the descending aorta. Consensus guidelines developed in 2009 suggest that ascending aortic aneurysms greater than or equal to 5.5 cm warrant surgical repair [3]. Surgery is not recommended for aneurysms less than 5 cm diameter. However even with a smaller diameter there is still a risk of complication. Thus even for MTAA patients with an initial aneurysm size of 4–4.9 cm, there is 5 - 10% yearly risk of rupture, dissection or death [12]. To-date, the sub-group of these MTAA patients at highest risk for a future adverse event has not been well established - driving the contemporaneous practice for risk factor modification and close surveillance. With increasing awareness of the danger of radiation exposure however, there remains a real need to identify an optimal surveillance interval differentially for follow-up of “at-risk” versus “not-at risk” MTAA patient sub-groups.

Given their inherent, asymptomatic nature, the true MTAA incidence cannot be known. However in an interesting study, Itani et al. reported the detection of asymptomatic aortic aneurysms in a mass lung cancer screening program using mobile helical computed tomography units. They found 11 of 6,971 (0.16%) of screened subjects to have aortic aneurysms [13]. Similarly in our study, all of our MTAA patients presented with asymptomatic aneurysms and were diagnosed as an incidental finding.

Of the 110 records reviewed, only 14 (13%) met the endpoint criteria of substantive growth or requiring surgery. There were no aortic aneurysm related deaths. Throughout our study period, the assessment and follow-up practices at our institution were the same for all study patients and followed current convention which among many practitioners has been to repeat imaging every 6 months up to 1-year; then once aneurysm stability has been established to perform annual imaging thereafter.

The very low aneurysm growth rate of our MTAA patients during the follow-up period suggests that such annual imaging of all patients, the unstated “standard of care”, may be excessive and unnecessary for “lower -risk” MTAA patient sub-groups’ as well as pose a potential health hazard related to excessive radiation exposure which in this study was not insignificant. Although the exact radiation doses could not be determined (due to missing CT radiation exposure data), there were 4.8 CT scans per patient with an estimated mean cumulative effective radiation dose of 34 mSv per patient.

Given difficulties of comparing aneurysm sizes across imaging modalities, the CT scan has been generally considered as the “gold standard” approach that should be used to measure aneurysm size. As low dose CT imaging becomes more refined, the future radiation exposure to patients being followed for aneurysm and other intra-thoracic pathology may decrease. Until low dose CT scans become routine, however, alternate imaging approaches should be further explored to ensure the most accurate, cost-effective and safe modality for MTAA patients and optimal interval of imaging needs to be clarified.

Unfortunately, many patients are not able to undergo alternative imaging approaches. Although MRI has less radiation exposure, MRI procedures are currently lengthy and expensive and the presence of a devices such as pacemakers renders them ineligible. While cheaper, safer, and more readily accessible, echocardiography appears to be a less reliable approach to document aneurysm size, as the echo findings may be (at least, in part) operator dependent [7]. Moreover, aneurysms of the arch and descending thoracic aorta cannot be accurately followed using an echo-based surveillance strategy, making the use of echo as the sole method of measurement of aortic size controversial. Hence, only CT scan findings were used for purposes of this pilot study to assess index and changes over time in aneurysm size.

Previous studies have established guidelines for when to operate on large aneurysms; defining a “hinge point” where the percentage point increase of complications is significantly amplified after a certain aortic diameter is exceeded. In our study, we used the negative likelihood ratio (as a diagnostic test performance metric) to identify a similar “hinge point” where MTAA patients were more likely to achieve the study endpoint based on their index aneurysm size. As the likelihood of an adverse event increases above the threshold of 4.3 cm, more frequent surveillance of “at-risk” patients might be warranted. Conversely “not-at-risk” patients may not need to be subjected to frequent imaging leading to significant amounts of radiation with unclear benefit.

This study is retrospective in nature, and therefore has several inherent limitations. While the total patient population size is low, it is comparable to other published studies reporting a unique patient sub-group. Measurements of the aortic size were derived from the radiology report and not directly measured from the scan. However since many surgeons depend on radiology reports and reported sizes for comparisons this data extraction method may be clinically relevant. Aortic diameter was not normalized to patients’ body-surface area in this analysis as current guidelines employ diameter in their recommendations. In future studies however, we think it would be meaningful to also analyze normalized aortic size to clarify the potential limitation of aortic diameter alone as predictor of risk. Finally, as radiation doses associated with the images performed at our VA Medical Center were not consistently or uniformly recorded, a recently published average CT radiation dosage per scan was used to estimate our patients’ average cumulative radiation exposure [11].

## Conclusion

As a very novel approach, the 4.3 cm index TAA threshold will require external validation, application, and evaluation in a different MTAA patient population. However, this study’s threshold has the potential to aid clinicians in identifying “at-risk” versus “not-at-risk” patient subgroups with MTAA. This is in contrast to the current strategy which is based on best clinical judgment. A data driven patient care plan will guide an optimal surveillance strategy for these patients and spare many the risk and exposure of unnecessary CT imaging.

## Abbreviation

MTAA: Moderate thoracic aortic aneurysms
